# Comparison of factor XII levels in gestational diabetes, fetal macrosomia, and healthy pregnancies

**DOI:** 10.1186/s12884-020-03455-0

**Published:** 2020-12-02

**Authors:** Esra Ozbasli, Ozguc Takmaz, Emine Karabuk, Mete Gungor

**Affiliations:** Department of Obstetrics and Gynecology, Acibadem Mehmet Ali Aydinlar University School of Medicine, Darüşşafaka, Büyükdere Cad. No: 40, Sarıyer, 34457 Istanbul, Turkey

**Keywords:** factor XII, pregnancy, gestational diabetes

## Abstract

**Background:**

If not detected and treated, gestational diabetes mellitus (GDM) can cause serious pregnancy complications such as macrosomia, preeclampsia, and fetal/neonatal mortality. Many studies have examined underlying contributing factors for GDM, including hypercoagulation. Factor XII (FXII) is a coagulation factor that increases throughout normal pregnancies, and we evaluated the relationship of GDM with FXII, FXIIa (activated FXII), and other coagulation parameter levels. GDM and macrosomia are closely related, but it is not known whether FXII could be an independent causal factor for macrosomia.

**Methods:**

In this prospective study, blood samples were taken from 69 pregnant women at the time of term delivery to determine levels of FXII, FXIIa, and other coagulation parameters. Based on the results, pregnancies fell into GDM, non-diabetic with macrosomia (M), or healthy (C [control]).

**Results:**

FXII concentration levels were significantly higher in GDM patients compared with the M and C groups. There were no significant differences when comparing FXIIa, activated partial thromboplastin time, prothrombin time (PT), and international normalized ratio. The GDM group saw a significant negative correlation between FXII concentrations and maternal pregestational body mass index (BMI) and BMI before delivery. In the M group, a positive correlation was observed between FXII concentrations and newborn weight and newborn weight percentile.

**Conclusions:**

An increase in FXII levels was observed in patients with gestational diabetes. Associations between coagulation parameters and GDM should be further analyzed to define the mechanisms of GDM and possible treatment modalities.

**Trial registration:**

Our study has been registered at clinicaltrials.gov (NCT03583216). Registered on July 11, 2018,

## Background

Gestational diabetes mellitus (GDM) is a common pregnancy complication [1], with a 2016 US prevalence of 6%, according to the Centers for Disease Control and Prevention [2]. The prevalence varies worldwide due to differences found in racial/ethnic groups, diagnostic criteria, and testing methods [3]. Adverse outcomes such as macrosomia, preeclampsia, and fetal/neonatal mortality can increase if GDM is not diagnosed and treated [4]. In GDM, elevated plasma coagulation activation markers are found (thrombin-antithrombin complex) and clotting factors (fibrinogen; factors VII, VIII, XI, and XII; kallikrein; and von Willebrand). While the pathogenesis and underlying molecular mechanisms are not well understood, findings support the clinical observation that GDM is a hypercoagulable state, with low-level inflammation and a higher level of thrombotic events [5–8].

Coagulation factors increase in the maternal plasma throughout normal pregnancy [9], which is necessary to stop the blood flow of up to 700 ml/min immediately after placental separation. Factor XII (FXII, Hageman factor) is part of the normal coagulation system and increases in pregnant women with each week of gestation [9, 10]. While FXII is intrinsically related to coagulation in normal pregnancy and is also a marker in GDM, there have been no studies to explore the role of FXII in GDM.

FXII induces inflammation [11], and FXIIa induces fibrin production in the intrinsic coagulation cascade and activates the kallikrein-kinin system [12]. The kallikrein-kinin system is activated in uncontrolled or poorly controlled diabetic individuals, which increases the level of FXII [13]. FXII shows mitogenic activity in a zymogenic form; this might be related to macrosomia [14, 15], which itself can have adverse fetal, neonatal, and even adult outcomes.

Animal studies show thrombosis decreases with FXII inhibitors [12, 16]. These studies describe drugs that selectively inhibit FXIIa, such as recombinant purely human antibody 3F7 and recombinant new infestin-4 [16, 17]. These drugs could prevent thrombosis without the risk of bleeding inherent with anticoagulants such as heparin.

We hypothesized that FXII levels might differ when comparing healthy pregnant women with those with GDM or macrosomic fetuses. We compared the pre-labor levels of FXII and other coagulation parameters among three groups of term pregnant women who had [i] GDM pregnancies (group GDM), [ii] non-diabetic fetal macrosomia (group M), or [iii] healthy pregnancies (control [group C]).

The study’s primary goal was to compare the FXII and FXIIa levels in GDM. The secondary goal was to evaluate FXII levels in macrosomia.

## Methods

This was a non-randomized, prospective cohort study conducted in a single institution, Acibadem Maslak Hospital, Istanbul, Turkey, from July to November 2018. It was approved by the Medical Ethics Committee of the Institutional Ethical Review Board of the Acibadem Mehmet Ali Aydinlar University School of Medicine Mehmet Ali Aydınlar Acibadem University School of Medicine (ATADEK ID no: 2017-10/2) and registered at clinicaltrials.gov (number: NCT03583216); written informed consent was obtained from all participants.

### Subjects

Sixty-nine term pregnant women with GDM, fetal macrosomia, or normal low-risk pregnancies were included in the study. Patients were excluded from the study if they were less than 18 years old, had a preterm delivery (< 37 weeks of pregnancy), post-term delivery (> 42 weeks of pregnancy), high-risk pregnancy (preeclampsia, intrauterine growth retardation, placenta previa, placenta accreta, or oligohydramnios), or a diagnosis of gestational or pre-gestational systemic diseases (hypertension, liver disease, kidney disease, pulmonary disease, hematologic disorders such as inherited coagulation defects, or gestational hypertension). When patients were admitted to the clinic, they were selected and included in the study based on their eligibility status. All data were obtained from their charts or from interviews during their deliveries at the hospital. Infant height and weight were calculated on the day of delivery by the pediatric nurse.

Patients were stratified into three groups: [i] GDM during pregnancy (*n* = 22), [ii] macrosomia during pregnancy (M group, n = 22), or [iii] no complications during pregnancy (C [control] group, *n* = 25). All participants were screened with a 50 g glucose tolerance test between 24 and 28 weeks of gestation. A plasma value of ≥ 140 mg/dl was a threshold for performing a three-hour oral glucose tolerance test (OGTT). GDM was confirmed by a 100 g glucose tolerance test if at least two of the Carpenter–Coustan criteria [10] were met: [i] fasting blood glucose ≥ 95 mg/dL, [ii] blood glucose ≥ 180 mg/dL in the test’s first hour, [iii] ≥ 155 mg/dL in the second hour, and [iv] ≥ 140 mg/dL in the third hour. Fasting was not required before the screening test, but 12 hours of fasting was requested before the three-hour OGTT. Patients in the GDM group were screened between 24 and 28 weeks of gestation and all met the Carpenter–Coustan criteria. These individuals managed to keep their glucose levels under control through diet and exercise.

Newborn macrosomia is defined as birth weight ≥90th percentile for gestational age [18]. Fetal macrosomia was detected during pregnancy and confirmed at our clinic upon delivery. Five patients in the postpartum period were excluded from the study because the weight of their newborn was not compatible with the macrosomia criteria. Patients with normal, uncomplicated pregnancies were recruited for the control group.

All procedures performed in studies involving human participants were under the ethical standards of the institutional and/or national research committee and with the 1964 Helsinki Declaration and its later amendments or comparable ethical standards. The study was approved by the Medical Ethics Committee of the Institutional Ethical Review Board of the Acıbadem MAA University School of Medicine (ATADEK ID no: 2017/10).

### Methods

Blood samples (4 ml) were taken just before delivery from the antecubital vein and stored in two vacutainer tubes with citrate sodium (3.8%). Room-temperature samples were centrifugated at 2500 rpm for 15 min to obtain platelet-poor plasma, which was frozen at − 80 °C. Samples received automated coagulation analysis using commercial kits to obtain concentration levels: FXII antigen level (ELISA kits), FXIIa (coagulation FVII deficient plasma), aPTT (Siemens Dade® Erlangen, Germany Actin® FS Activated PTT Reagent), and PT (Siemens Thromborel® S). The concentration ranges were 1.5–300 ng/ml for FXII (sensitivity: 1.366 ng/ml) and 58–166% for FXIIa.

### Statistical analysis

Descriptive statistics are presented as frequency, percentage, median, and mean ± standard deviation. Testing for normality was performed with the Shapiro–Wilk test, capturing skewness and kurtosis values using q-q plot graphs. A Fisher’s exact test or Pearson chi-square test was used to analyze categorical data. The relationship of numerical data was evaluated with a Spearman correlation test. One-way analysis of variance was used when assumptions of normal distribution and variance homogeneity were met in the measurements for more than two groups; Tukey’s test assessed binary comparisons. Welch’s test was used when assumption of normal distribution was assured; Tamhane’s test assessed binary comparisons. When the normal distribution assumption was not provided, the Kruskal–Wallis test was used; Bonferroni–Dunn procedure assessed binary comparisons. Statistical significance was set at* P* < 0.05. Analyses were made with R Version 3.5.2 [R Core Development, 2018).

## Results

**Fig. 1:**
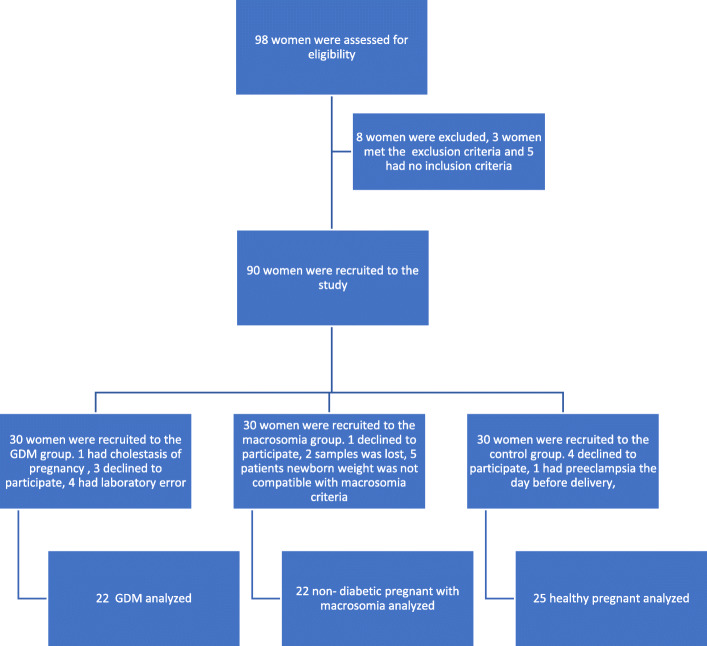
Patient flow chart from recruitment to study completion

Ninety-eight women admitted to our obstetrics and gynecology clinic for delivery from July to November 2018 were assessed for study recruitment, and 69 met the inclusion criteria: 22 in the GDM group, 22 in the M group, and 25 in the control group (Fig. 1). The groups had comparable data for mean age, parity, multiparity, maternal height, maternal BMI before pregnancy, maternal weight gain, and gender of the infant (*p* > 0.05) (Table 1). The M group had significantly higher gestational age at delivery, infant weight, infant weight percentile, cesarean section rate, and maternal BMI before delivery (*p* < 0.05). The GDM and C groups had comparable cesarean section rates (*p* > 0.05).

**Table 1:** Demographic features of the groups

	CONTROL (*n* = 25)	GDM (*n* = 22)	MACROSOMIA (*n* = 22)	*p-value*
Age (years)	31.6 ± 4.03	32.55 ± 2.22	30.5 ± 4.01	^1^0.168
Parity (%)				
Multipara	7 (28)	8 (36.4)	7 (31.8)	^4^0.828
Primipara	18 (72)	14 (63.6)	15 (68.2)	
Gestational age at delivery (weeks) Median (Q1–Q3)	38.6(38.2–39.2)	38.8(38.4–39.3)	39.05(38.4–40)	^3^0.001**
Maternal Height (cm)	166.28 ± 5,32	164.32 ± 5,18	168 ± 5.72	^1^0.085
Pre-gestational BMI (kg/m^2^)	22.36 ± 2.73	23.5 ± 4.61	25.05 ± 5.12	^3^0.276
BMI before delivery (kg/m^2^)	27.46 ± 2.69	28.7 ± 5.28	30.97 ± 5.11	^1^0.022*
Maternal weight gain (kg)	14 ± 2.84	14.18 ± 3.66	16.45 ± 5.62	^3^0.186
Infant’s gender (%)				
Female	13 (52)	11 (50)	13 (59)	^2^0.816
Male	12 (48)	11 (50)	9 (41)	
Infant height (cm)	49.84 ± 1,28	49.5 ± 1.74	51.18 ± 1.37	^3^0.001**
Infant weight (gm)	3183.6 ± 287.37	3325.23 ± 480.53	3931.82 ± 256.3	^3^<0.001**
Infant weight (% percentile)	57.8 ± 19.64	66.09 ± 27.92	96 ± 3.28	^3^<0.001**
Delivery method (%)				
Cesarean section	19 (76)	18 (81.8)	22 (100)	^4^0.044
Vaginal delivery	6 (24)	4 (18.2)	0 (0)	
Indication for cesarean (%)				
Repeat cesarean	5 (20)	8 (36)	7 (32)	
Maternal demand	1 (4)	0 (0)	1 (5)	
Malpresentation	5 (20)	2 (9)	1 (5)	
Macrosomia	0 (0)	1 (5)	5 (22)	
CPD	1 (4)	4 (18)	4 (18)	
Others	13 (52)	7 (32)	4 (18)	

Values are mean ± SD, median (Q1–Q3) or n (%) of cases in a group,

*SD Standard deviation, Q1 25th percentile, Q3 75th percentile*

*p < 0.05, **p < 0.01

^1^ANOVA F test, ^2^ Pearson chi-square test, ^3^ Kruskal–Wallis H test, ^4^ Fisher’s eExact test

**Fig. 2:**
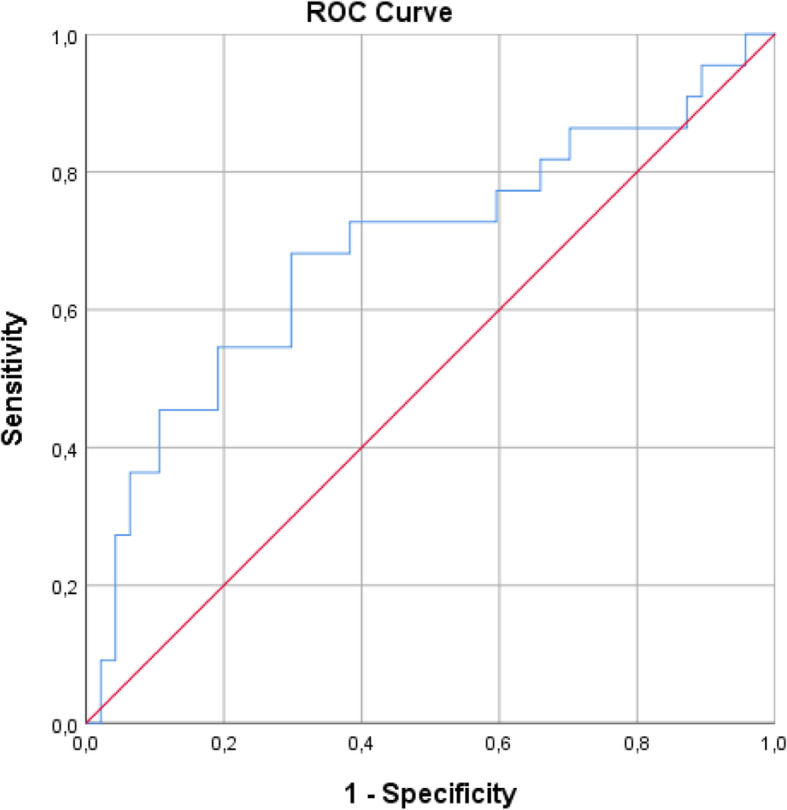
ROC Curve of gestational diabetes mellitus for prediction of high/low levels of FXII

The mean FXII concentrations of the GDM, M, and C groups were 93.9 ± 38.52 ng/ml, 78.9 ± 33 ng/ml, and 72.7 ± 23.24 ng/ml, respectively. The mean FXII concentration level of the GDM group was significantly higher than the control and M groups (*p* = 0.029). The M group had a higher FXIIa level than the GDM and control groups (119.88 ± 33.46%, 116.58 ± 26.41%, and 117.45 ± 57.54%, respectively), but there was no significant difference (*p* = 0.521). The groups had no significant differences when comparing aPTT, PT, PT activity percentage, and INR levels (Table 2). A receiver operating characteristic curve was obtained to predict high/low levels of FXII concentrations from GDM status, and the area under the curve was 0.68 (95% confidence interval: 0.54–0.83, *p* = 0.01). A threshold of 77.4 had a sensitivity of 68% and specificity of 70% (Fig. 2).

**Table 2:** **−** Haemostatic parameters of controls, GDM and non− diabetic pregnant with macrosomia

	CONTROL (*n* = 25)	GDM (*n* = 22)	MACROSOMIA (*n* = 22)	*p-value*
FXII Conc. (ng/ml)	72,7 ± 23,24	93,9 ± 38,52	78,9 ± 33	0,029*
FXII Act. (%)	117,45 ± 57,54	116,58 ± 26,41	119,88 ± 33,46	0,521
APTT (sn)	25,01 ± 2,03	24,93 ± 1,54	24,98 ± 2,33	0,900
PT (sn)	10,19 ± 0,44	10,33 ± 0,44	10,42 ± 0,55	0,240
PT activity %	114,6 ± 9,06	109,88 ± 11,31	109,07 ± 12,24	0,173
INR	0,93 ± 0,05	0,95 ± 0,06	0,95 ± 0,05	0,619

Data was presented mean ± standard deviation

In the GDM patients, A negative correlation was observed between FXII concentrations in the patients’ pre-gestational BMI (*r* = − 0.551, *p* = 0.008) and BMI before delivery (*r* = − 0.513, i = 0.015). In the M group, a positive correlation was observed between FXII concentrations, infant weight (*r* = 0.441, *p* = 0.04), and infant weight percentile (*r* = 0.60, *p* = 0.003); a negative correlation was observed between FXII concentrations, BMI before pregnancy (*r* = − 0.443, *p* = 0.039), and BMI before delivery (*r* = − 0.531, *p* = 0.011). In the C group, a moderately negative correlation was observed between FXII concentrations and INR (*r* = − 0.464, *p* = 0.02). A moderate negative correlation was observed between FXIIa and aPTT (*r* = − 0.482, *p* = 0.015).

We note that although the sample size of our study was calculated before it began, the study’s power was determined after it finished. In a post-hoc power analysis for FXII concentration between GDM, M and C groups with the F-test formula of the G-Power 3.1.9.3 program (Heinrich Heine Universität Dusseldorf, 2017), the power of the study was found to be 80% with an effect size of 0.38 at an α-level of 0.05.

## Discussion

This study compares FXII levels in GDM, non-diabetic pregnancies with macrosomia, and healthy pregnancies. FXII is a coagulation factor that plays a role in the activation of the intrinsic clotting pathway, and Patrassi et al. suggest there is enhanced activation of the intrinsic clotting system in patients with diabetes mellitus [13, 15]. There is a hypercoagulable state in diabetes, and FXII is one of the coagulation factors shown to be increased [7, 8, 13].

Endothelial damage due to hyperglycemia can activate the intrinsic coagulation system [19]. It has been reported that FXII increases during pregnancy [10, 20], but, to our knowledge, no studies on plasma levels of FXII in GDM patients had been reported. In our study, we found that women with GDM have significantly higher levels of FXII compared to women with healthy pregnancies (p < 0.05).

There are studies suggesting that zymogen FXII may exhibit growth factor-like (EGF- like) activities [11, 15]. This EGF- like “domain” of FXII can regulate liver growth and enhance protein production in the liver, which may stimulate the production of FXII and possibly other coagulation factors. This growth factor-like activity of FXII might be the source of the underlying pathology of hypercoagulability and adverse outcomes such as preeclampsia, macrosomia, birth trauma, and stillbirth in GDM. Although we did not detect any significant increases in FXII levels in the non-diabetic macrosomia group, there was a positive correlation between FXII levels and infant weight. This suggests that investigating the effects of FXII’s zymogen is beneficial, as mentioned in previous studies [11, 15].

aPTT might also have a relationship with hypercoagulability. Hron et al. showed that a shortened aPTT might be a marker for hypercoagulability [21], and Lippi et al. concluded those with impaired fasting glucose and diabetes displayed significantly shortened aPTT [22]. In our study, there was no significant difference in aPTT levels in any groups. This was attributed to all of our patients, including the GDM group, being euglycemic.

Our study saw a negative correlation between FXII concentrations, aPTT, and INR in the control group: as FXII concentrations increased, aPTT and INR decreased (r = − 0.482, p = 0.015 and r = − 0.464, p = 0.02, respectively). Conversely, another study showed a negative correlation between FXII deficiency and aPTT prolongation [23]. This finding suggests that FXII inhibitors could be a promising treatment for thrombosis.

We saw no statistically significant increase in FXII levels in the non-diabetic macrosomia group. However, there was a positive correlation between FXII levels and infant weight and weight percentile, meaning that infant weight increased with higher levels of FXII (r = 0.60; p = 0.03). This suggests a role for FXII in macrosomia. FXII inhibitors may be a promising treatment for preventing macrosomia and its associated complications.

Patrassi et al. found no correlation between the levels of blood glucose and those of prekallikrein, kallikrein, FXII, and FXIIa. Therefore, they concluded that the reduction of blood glucose levels would not decrease these parameters [14]. Thus, FXII inhibitors may be evaluated to treat the hypercoagulable state present in GDM [16, 17, 24].

There were limitations to our study. One of them was the small sample size, even though the power of the study is reasonable. More prospective studies with a larger sample size are needed to further confirm the association of FXII with GDM. In addition, concentrations of other thrombotic risk factors such as factor V Leiden, protein C, FXI, antithrombin III, platelets, and fibrinogen were not studied.

## Conclusions

We showed that FXII levels increase in the GDM group, which could help to explain one of the underlying complex mechanisms causing GDM. In addition, FXII inhibitors could prevent the pathogenesis and adverse outcomes observed in GDM. More prospective studies with larger populations are needed to confirm the effect of FXII in the pathogenesis of GDM.

## Data Availability

The datasets used for the current study are available from the corresponding author, per reasonable request.

## Abbreviations

GDM: Gestational diabetes mellitus
FXII: Factor XII
FXIIa: Activated FXII
M: Macrosomia
C: Control
PT: Protrombin time
BMI: Body mass index
US: United States
OGTT: Oral glucose tolerance test
MAA: Mehmet Ali Aydınlar
aPTT: Activated partial tromboplastin time
INR: International normalized ratio
FXIIconc.: Factor XII concentrations
Et al.: Et alia (and others)
EGF-like: epidermal growth factor- like
ROC: Receiver operating characteristic

## References

[1] Coustan DR (2013). Gestational diabetes mellitus. Clin Chem.

[2] Deputy NP, Kim SY, Conrey EJ, Bullard KM (2018). Prevalence and changes in preexisting diabetes and gestational diabetes among women who had a live birth - United States, 2012–2016. MMWR Morb Mortal Wkly Rep.

[3] Zhu Y, Zhang C (2016). Prevalence of gestational diabetes and risk of progression to Type 2 diabetes: a global perspective. Curr Diab Rep.

[4] Hussain SA, Smith AM, Cross JA (2020). Diabetes, fetal demise, and shoulder dystocia: the importance of glucose screening to prevent catastrophic obstetric outcomes. Case Rep Obstet Gynecol.

[5] Gallimore MJ, Harris SL, Jones DW, Winter M (2004). Plasma levels of factor XII, prekallikrein and high molecular weight kininogen in normal blood donors and patients having suffered venous thrombosis. Thromb Res.

[6] Teliga-Czajkowska J, Sienko J, Zareba-Szczudlik J, Malinowska-polubiec A, Romejko-Wolniewicz E, Czajkowski K (2019). Influence of glycemic control on coagulation and lipid metabolism in pregnancies complicated by pregestational and gestational diabetes mellitus. Adv Exp Med Biol.

[7] Barillari G, Fabbro E, Pasca S, Bigotto E (2009). Coagulation and oxidative stress plasmatic levels in a type 2 diabetes population. Blood Coagul Fibrinolysis.

[8] Carr ME (2001). Diabetes mellitus. J Diabetes Complications.

[9] O’Riordan MN, Higgins JR (2003). Haemostasis in normal and abnormal pregnancy. Best Pract Res Clin Obstet Gynaecol.

[10] Carpenter MW, Coustan DR (1982). Criteria for screening tests for gestational diabetes. Am J Obstet Gynecol.

[11] Renné T, Schmaier AH, Nickel KF, Blombäck M, Maas C (2012). In vivo roles of factor XII. Blood.

[12] Kenne E, Nickel KF, Long AT, Fuchs TA, Stavrou EX, Stahl FR (2015). Factor XII: a novel target for safe prevention of thrombosis and inflammation. J Intern Med.

[13] Patrassi GM, Vettor R, Padovan D, Girolami A (1982). Contact phase of blood coagulation in diabetes mellitus. Eur J Clin Invest.

[14] Cool DE, Edgell CJS, Louie GV, Zoller MJ, Brayer GD, MacGillivray RT (1985). Characterization of human blood coagulation factor XII cDNA. Prediction of the primary structure of factor XII and the tertiary structure of β-factor XIIa. J Biol Chem.

[15] Schmeidler-Sapiro KT, Ratnoff OD, Gordon EM (1991). Mitogenic effects of coagulation factor XII and factor XIIa on HepG2 cells. Proc Natl Acad Sci U S A.

[16] Xu Y, Cai TQ, Castriota G, Zhou Y, Hoos L, Jochnowitz N (2014). Factor XIIa inhibition by Infestin-4: in vitro mode of action and in vivo antithrombotic benefit. Thromb Haemost.

[17] Kolyadko VN, Lushchekina SV, Vuimo TA, Surov SS, Ovsepyan RA, Korneeva VA (2015). New Infestin-4 mutants with increased selectivity against Factor XIIa. PLOS ONE.

[18] Duryea EL, Hawkins JS, McIntire DD, Casey BM, Leveno KJ (2014). A revised birth weight reference for the United States. Obstet Gynecol.

[19] Lemkes BA, Hermanides J, Devries JH, Holleman F, Meijers JCM, Hoekstra JBL (2010). Hyperglycemia: A prothrombotic factor?. J Thromb Haemost.

[20] Briseid K, Hoem N-O, Johannesen S, Fossum S (1991). Contact activation factors in plasma from pregnant women–increased level of an association between factor XII and kallikrein. Thromb Res.

[21] Hron G, Eichinger S, Weltermann A, Quehenberger P, Halbmayer WM, Kyrle PA (2006). Prediction of recurrent venous thromboembolism by the activated partial thromboplastin time. J Thromb Haemost.

[22] Lippi G, Franchini M, Targher G, Montagnana M, Salvagno GL, Guidi GC (2009). Epidemiological Association between fasting plasma glucose and shortened APTT. Clin Biochem.

[23] Gorar S, Alioglu B, Ademoglu E, Uyar S, Bekdemir H, Candan Z (2016). Is there a tendency for thrombosis in gestational diabetes mellitus?. J Lab Phys.

[24] Weitz JI (2016). Factor XI and factor XII as targets for new Anticoagulants. Thromb Res.

